# Differences in cooking taste and physicochemical properties between compound nutritional rice and common rice

**DOI:** 10.3389/fnut.2024.1435977

**Published:** 2024-07-31

**Authors:** Dongmeng Zhang, Jian Li, Dongping Yao, Jun Wu, Qiuhong Luo, Hong Shen, Meixia Hu, Fudie Meng, Ying Zhang, Xionglun Liu, Yang Shan, Dongbo Liu, Bin Bai

**Affiliations:** ^1^State Key Laboratory of Hybrid Rice, Hunan Hybrid Rice Research Center, Hunan Academy of Agricultural Sciences, Changsha, China; ^2^College of Agronomy, Hunan Agricultural University, Changsha, China; ^3^College of Horticulture, Hunan Agricultural University, Changsha, China; ^4^Hunan Provincial Engineering Research Center of Medical Nutrition Intervention Technology for Metabolic Diseases, Changsha, China; ^5^Central Region, Regional Nutrition Innovation Platform, Changsha, China; ^6^College of Plant Science and Technology, Hunan Biological and Electromechanical Polytechnic, Changsha, China; ^7^College of Advanced Agricultural Sciences, Zhejiang A & F University, Hangzhou, China

**Keywords:** compound nutritional rice, steamed food quality, rice nutrition, extrusion, physicochemical properties

## Abstract

In this study, it was compared the physicochemical properties and cooking taste quality between four different types of compound nutritional rice (rice flour with the addition of other coarse grains, legumes, potatoes, and other powders, extruded as artificial rice grains) and common rice. We found that the protein and apparent amylose contents of compound nutritional rice were higher than that of common rice, up to 9.775% and 19.45% respectively. The γ-aminobutyric acid (GABA) and resistant starch contents were much lower than in common rice, and the dietary fiber content did not differ from that in common rice. The results of Fourier transform infrared spectroscopy (FTIR) and X-ray diffraction (XRD) analysis revealed that the starch properties and structure of the compound nutritional rice changed due to high temperature and high pressure processing. In particular, the crystalline structures of starch became V-shaped. In addition, the results of artificial tasting and tasting meter showed that the taste of compound nutritional rice was generally inferior to that of common rice. In summary, compound nutritional rice had problems such as nutritional imbalance and poor taste. There was still a lot of room for improving the taste quality of compound nutritional rice. Therefore, the future development of compound nutritional rice should focus on both nutritional balance and taste improvement. The results of this paper also provided a certain theoretical basis for this.

## 1 Introduction

As one of the major food crops, rice is a staple food for 50% of the world’s population (1). Rice is rich in numerous nutrients, such as starch, protein, fat, and vitamins. In particular, starch, which is formed by amylose and amylopectin, is the most important part of rice, where amylose has a greater influence on the flavor quality of steamed rice (2). In general, the higher the amylose content, the poorer the taste quality of the rice, the faster the retrogradation speed, and the poorer its palatability. Protein content is the second-largest component of rice, and its influence on the flavor quality of steamed rice is also significant (3). Studies have shown that protein content is usually negatively correlated with rice steaming palatability quality (4).

The rice is processed into polished rice, which involves several procedures such as hulling, grinding, milling, and polishing. During processing, about 70% of the nutrients in the rice grain are lost. In particular, the content of trace elements which are considered daily needs for people is significantly reduced due to operations such as hulling and milling. Therefore, the concept of rice fortification has come into being. At the early stage, the main methods of nutritional fortification for rice are leaching and absorption, spraying, and powder fortification (5, 6); however, all three methods have the disadvantages of difficult operation, small audiences, and difficulty in promotion. Later, the extrusion nutritional fortification method became the mainstream method of nutritional fortification with the development of technology. Compared with other methods, the extrusion nutritional fortification method has the advantages of simple operation, easy digestion, and less regeneration (7). The extrusion nutritional fortification method involves the creation of rice by mixing and extruding starchy raw rice flour with nutritional fortification substances, such as bean flour, vegetable flour, and cordyceps flour, through a series of operations including mixing, extrusion, cutting, and drying. The finished product is called compound nutritional rice, also known as reconstituted rice or extruded rice, among others. At present, compound nutritional rice has good prospects, as its efficacy can be adjusted according to demand (8).

Due to recent changes in people’s diets, the increased intake of high-carb, high-calorie foods has increased the probability of obesity and insulin resistance (9). Over the last 15 years, economic spending related to diabetes has increased by about 316%, which is enough to see a dramatic increase in the number of diabetics in the world. As a result, a number of products suitable for diabetics have emerged (10). White rice has a high glycemic index (GI) (11), and a low GI compound nutritional rice developed for diabetics on the market can effectively slow the increase in blood sugar in the body when eating this product. Regarding compound nutritional rice, studies have shown that the process of extruding rice flour into rice at high temperatures causes changes in its starch structure, function, and physicochemical properties (7), and that the nutritional quality of cooked rice depends on its physicochemical properties (12). In addition, the addition of bean powder, vegetable powder, and cordyceps powder to rice flour can effectively increase its nutritional composition and nutrient content. The application of compound nutritional rice provides a solid foundation for precision nutrition and personalized nutrition treatment.

However, we do not yet have a clear understanding of the specific differences between compound nutritional rice and common rice. Therefore, the purpose of this study was to compare four types of compound nutritional rice, which sell better in the market, using high-quality rice as a control, in order to investigate the differences between the physicochemical properties, starch structure, and steaming quality between compound nutritional rice and common rice, aiming to promote improvements in the nutritional composition and steaming characteristics of compound nutritional rice.

## 2 Materials and methods

### 2.1 Materials

Yuzhenxiang polished rice (YJ), Yuzhenxiang brown rice (YC), compound nutritional Rice No. 1 (CNR1), compound nutritional Rice No. 2 (CNR2), compound nutritional Rice No. 3 (CNR3), and compound nutritional Rice No. 4 (CNR4) were tested. YJ & YC were obtained from Hunan Academy of Agricultural Sciences, Changsha, Hunan 410125, China. The nutrient framework for the four kinds of compound nutritional rice was similar, with rice as the basic raw material (a content of 70%), supplemented with the addition of a total amount of about 10% of millet, corn, buckwheat, oat, quinoa, and other cereal crop flours; a total amount of about 10% of purple yam, sweet potato, potato, and other potato flours; about 5% of bitter gourd, spinach, pumpkin, yam, kudzu root, goji berry, konjac, and other nutritious vegetable powders; and about 5% of legume and Chinese herbal medicine (e.g., chrysanthemum and poria). Note that the nutrient powders in each major category were added in equal proportions. Compared with CNR1, CNR2 had an increased content of Luo Han Guo and chrysanthemum by about 1.5% and a relatively reduced content of purple yam, sweet potato, and bitter gourd; CNR3 had an increased content of wheat, lily, and so on by about 1%; and finally, soy protein powder was replaced with Highland barley in CNR4. In addition, there were differences in the proportions of raw material ingredients in all the compound nutritional rice, and there was some randomness in the selection of raw materials. Please refer to the Supplementary Table S1. The process of making the compound nutritional rice involved three stages at high temperatures: 65°C, 108°C, and 85°C. Specific preparation is as follows: all kinds of raw materials powder according to a certain proportion of mixing, adding water for the total mixed powder mass of 21%, high-speed mixing for 10 min, immediately into the Twin Screw Extruder (DS32-II, China) for extrusion. Extruded products immediately hot air drying and dehydration, the final product for the compound nutritional rice.

### 2.2 Nutrient chemical content determination

Determination of 17 amino acids was conducted using an automatic amino acid analyzer (LA8080, Hitachi, Japan). Specific methods referred to the national standard GB 5009.124-2016 of the People’s Republic of China.

The protein content was determined according to the national standard GB 5009.5-2016 of the People’s Republic of China.

The dietary fiber content was determined according to the national standard GB5009.88-2014 of the People’s Republic of China.

For determination of GABA (γ-aminobutyric acid), the sample was ground into powder and sieved (100 mesh). A 0.1 g sample was accurately weighed into a centrifuge tube, an equal volume of 0.1 mol/L pre-cooled PBS solution was added, the solution was thoroughly mixed and centrifuged at 2,500 r/min, and the supernatant was set aside and the volume was fixed to 1 mL. The liquid was reserved for subsequent assays. After equilibration for 20 min at room temperature, the desired slides were removed from the aluminum foil bag and the remaining slides were returned to 4°C in a sealed self-sealing bag. The standard wells and sample wells were then set up by adding 50 μL of standards of different concentrations to each of the standard wells, then adding 10 μL of the sample to be tested and 40 μL of the sample dilution to the sample wells, making sure that the blank wells were not loaded. Except for the blank wells, 100 μL of horseradish peroxidase (HRP)-labeled detection antibody was added to each well of the standard and sample wells. The reaction wells were sealed with sealing film, and then incubated in a 37°C water bath or thermostat for 60 min. The liquid was then discarded and dried on blotting paper, and each well was filled with washing solution. The set-up was allowed to stand for 1 min and the washing solution was shaken off and patted dry on blotting paper. The washing step was repeated 5 times (the plate can also be washed with a plate washer), after which 50 μL each of substrate A and B was added to each well, then incubated at 37°C for 15 min. Then, 50 μL of termination solution was added to each well, and the OD value of each well at 450 nm was measured within 15 min.


$$\mathrm{GABA}\;\left(\mu g/g\right)=5\times C\times V÷M÷1000.$$

### 2.3 Aromatic compounds (2-AP) determination

The sample fragrance was analyzed by gas chromatography-mass spectrometry (GCMS-QP2020 NX SHIMADZU). The steps were as follows: 0.1 g of the fine rice powder sample (passed through a 200 mesh sieve) was weighed in a jaw bottle, then 2 mL of extraction reagent (absorb 10 mg/L TMP solution to 5 mL, and mix anhydrous ethanol and dichloromethane (1:1 volume ratio) to 100 mL) was added. The bottle was tightly sealed and placed in a water bath at 80°C for 3 h. It was then removed and allowed to cool down to room temperature, and was passed through a 0.22 μm filter membrane and transferred to the injection bottle for measurement. Chromatographic conditions: the column was an SH-Rxi-5Sil MS (30 m × 0.25 mm × 0.25 μm; Shimadzu, Japan), with a column temperature ramp-up procedure of 50°C for 2 min, and then ramped up to 280°C at 10°C/min for 3 min. The inlet temperature was 170°C, the carrier gas was high purity (purity >99.999%) helium, and the injection mode was split injection. Mass spectrometry conditions: electron bombardment (EI) ion source, ion source temperature of 230°C, and interface temperature of 280°C. The sample volume was 1 μL. Phytohormone standards of 5 ng/mL, 10 ng/mL, 25 ng/mL, 50 ng/mL, and 100 ng/mL were used to obtain the mass spectrometry peak intensity of the corresponding quantitative signals at each concentration of standards. The peak intensity of the corresponding quantitative signal at each concentration of the standard was obtained, the concentration (ng/mL) of the standard was used as the horizontal coordinate, and the peak area was used as the vertical coordinate to plot the standard curve.

### 2.4 Determination of apparent amylose content and resistant starch content

The apparent amylose content (AAC) was determined according to the agricultural industry standard NY/T 2639-2014 of the People’s Republic of China; we refer the reader to a previous study (13) for the specific experimental steps. The resistant starch content was determined using a resistant starch detection kit (Megazyme, K-RSTAR).

### 2.5 X-ray diffraction measurement

An X’Pert Pro X-ray diffractometer (PANalytical, Netherlands) was used for the X-ray diffraction (XRD) analysis. The starch samples were scanned in the range of 5°–60° with a step size of 0.02° and a scan speed of 4°/min. The data were analyzed using the MDI Jade 5.0 software (Materials Data, California, United States). The Degree of crystallinity (%), crystalline morphology, and diffraction peaks at 2θ value (angle) parameters were calculated separately.

### 2.6 Fourier transform infrared spectroscopy determination

A Nicolet iZ-10 Fourier transform infrared spectrometer with 32 scans and an instrumental resolution of 4.00 cm^−1^ was used to determine the ratio of ordered to disordered structures in starch granules.

### 2.7 Water absorption index and water solubility index determination

According to a literature method with minor changes (14), the samples were crushed and passed through 100 mesh sieve. Then, 2.5 g (*m*_0_) of sample powder was accurately weighed into a dry weighed centrifuge tube (*m*_1_), 30 mL of distilled water was added, the solution was stirred until completely dispersed and then held in a 30°C water bath for 30 min, taking it out and shaking every 10 min to keep the solution in suspension as much as possible. After 30 min, it was centrifuged at 1,006 × g for 15 min to separate the supernatant and precipitate. The supernatant was poured into a dry weighing aluminum box (*m*_2_) and dried and weighed (*m*_3_). The centrifuge tube from which the supernatant was poured and the precipitate in the tube (*m*_4_) were weighed at the same time.

The water absorption index (WAI) and water solubility index (WSI) were calculated as follows: WAI = *m*_4_ − *m*_1_/*m*_0_; WSI = *m*_3_ − *m*_2_/*m*_0_.

### 2.8 Pasting property measurement

The pasting properties of starch were analyzed using a Rapid Visco-Analyzer (RVA; Model RVA Super 4; Newport Scientific, Australia). The test profile was analyzed according to a previously described method (15).

### 2.9 Steamed taste quality determination

The flavor values of rice varieties were determined using a Rice Tastemeter (STA1B, SATAKE, Japan). First, 30 g of sample was weighed in a stainless steel tank, water was added, and the sample was soaked for 30 min. Stainless steel irrigation was then connected to the rice washing device and the sample was rinsed with water for about 30 s until the water flow was clear. For the control group (YJ & YC), water was added according to a rice: water ratio of 1:1.3, whereas the four compound nutritional rice had a rice: water ratio of 1:1. The stainless steel tank was covered with filter paper, sealed with a rubber sleeve, and put into a rice cooker to cook for 30 min, held for 10 min, and cooled in a cooler for 20 min. Then, 7 g of rice was weighed and compressed to a constant volume, and the composite flavor value of the rice was determined using a taste meter. Afterward, the rice cake compressed from 7 g of rice was used to determine the viscosity and hardness of the rice using a Hardness Viscometer (RHS1A, SATAKE, Japan).

### 2.10 Artificial flavor tasting

The rice was prepared according to the national standard GB/T 15682-2008 of the People’s Republic of China and scored by eight professionally trained researchers. Please see Supplementary Table S2 for specific judging rules.

### 2.11 Statistical analysis

All results were obtained in triplicate and expressed as mean ± SD. One-way analysis of variance (ANOVA, SPSS 23.0 SPSS Inc., Chicago, IL, United States) and Tukey’s tests were used to determine the evident difference between the variables. The difference between variables was significant when *p* < 0.05. All parameters shown in the tables and figures given in this article are the averages of the test data obtained from three replicates of each parameter for six samples.

## 3 Results and discussion

### 3.1 Nutrient chemical content and aromatic compounds content (2-AP)

The differences in nutrient chemical composition between the compound nutritional rice and the common rice are shown in Table 1. Except for CNR1 and CNR2, the moisture content of all varieties significantly differed. Among them, CNR3 had the highest moisture content, while YC had the lowest. In addition, overall, the moisture content of the compound nutritional rice was significantly higher than that of common rice. The reason for this was that, on one hand, rice flour was the main body of the experimental group, following which vegetable, bean, and other nutrient powders were added to form rice grains; the vegetable and other nutrient powders contain water themselves, which led to an overall increase in moisture content of rice grains. On the other hand, during the extrusion process, extrusion provided starch polymer shear force which, in turn, caused the starch structure to become loose and starch water absorption was enhanced, thus increasing the overall water content (16).

**Table 1:** Nutrient chemical composition of the six rice samples.

Samples	Moisture (%)	Protein (%)	2-AP (μg/kg)	GABA (μg/g)	Water-soluble dietary fiber (g/100 g)	Water-insoluble dietary fiber (g/100 g)
YJ	11.87 ± 0.15d	7.090 ± 0.062f	414.08 ± 46.49a	7.90 ± 0.10a	0.395 ± 0.077a	0.606 ± 0.092a
CNR1	14.10 ± 0.10b	8.015 ± 0e	N.D.	3.37 ± 0.11f	0.412 ± 0.081a	0.607 ± 0.117a
CNR2	13.73 ± 0.06b	9.775 ± 0.043a	N.D.	6.36 ± 0.11c	0.408 ± 0.075a	0.606 ± 0.089a
CNR3	16.63 ± 0.67a	8.385 ± 0.051c	N.D.	4.40 ± 0.13e	0.419 ± 0.023a	0.630 ± 0.017a
CNR4	12.53 ± 0.15c	8.660 ± 0.049b	N.D.	5.80 ± 0.12d	0.428 ± 0.029a	0.631 ± 0.050a
YC	11.23 ± 0.06e	8.320 ± 0.028d	511.56 ± 17.86a	7.23 ± 0.12b	0.419 ± 0.020a	0.612 ± 0.037a

Data are shown as the mean ± standard error of triplicate measurements. Different letters are followed after standard deviation to express significantly different (*p* < 0.05).

Significant differences in protein content existed among the six samples, with YJ having the lowest protein content and CNR2 having the highest. Comparing the common rice with the four experimental group samples, it was found that the various nutrients added to the compound nutritional rice resulted in a significant increase in the overall protein content of the rice grains. However, it is generally believed that the higher the protein content, the lower the steaming viscosity of cooked rice and the poorer the taste (17). Therefore, although the CNR2 variety had the highest protein content, it had poor eating sensation after steaming.

2-AP was determined for the six samples using gas chromatography-mass spectrometry (GC-MS). Among them, only common rice YJ and YC measured values, while the other four types had no 2-AP. The 2-AP values of YC were higher than those of YJ. According to a previous study (18), the 2-AP of rice is mainly produced by genetic and environmental influences. As the compound nutritional rice was reconstituted rice, its internal genetic structure was destroyed and, at the same time, the recombinant added nutritional powder also caused the flavor to disappear. It is known, from the literature, that GABA (also known as γ-aminobutyric acid) is a non-protein amino acid that is currently considered an antidepressant neurotransmitter (19). According to a previous study (20), a certain amount of GABA consumed by the human body through food facilitates various physiological functions such as improving sleep, improving depression, lowering blood pressure, and increasing protein secretion in the brain. In this study, there was a significant difference in GABA content among the six samples: the lowest content was found in CNR1, while the content in YJ was significantly higher than that of the other five samples. Overall, the GABA contents of compound nutritional rice were all significantly lower than those of common rice. The reason for this might be that glutamate dehydrogenase as well as glutamate decarboxylase were missing, and it is generally believed that, in rice, GABA is mainly converted from glutamate to GABA by the combined action of glutamate decarboxylase as well as glutamate dehydrogenase. However, as the compound nutritional rice is artificial rice, its internal enzyme activity is extremely low, thus hindering the synthesis of GABA and resulting in a significantly lower GABA content than that in the control group.

Dietary fiber can be classified according to its water solubility: water-soluble dietary fiber and water-insoluble. Although it is not digested and absorbed by enzymes in the human intestine, it is an important component of the diet (21). Due to the increasing refinement of national staple foods and the increasing number of patients with chronic diseases caused by dietary problems, increasing the daily intake of dietary fiber (among others) has become one of the ways to prevent the development of chronic diseases (22). Therefore, the level of dietary fiber content in compound nutritional rice is particularly important. However, in the present study, both dietary fibers did not significantly differ between the six samples, and its content was very similar.

**Figure 1:**
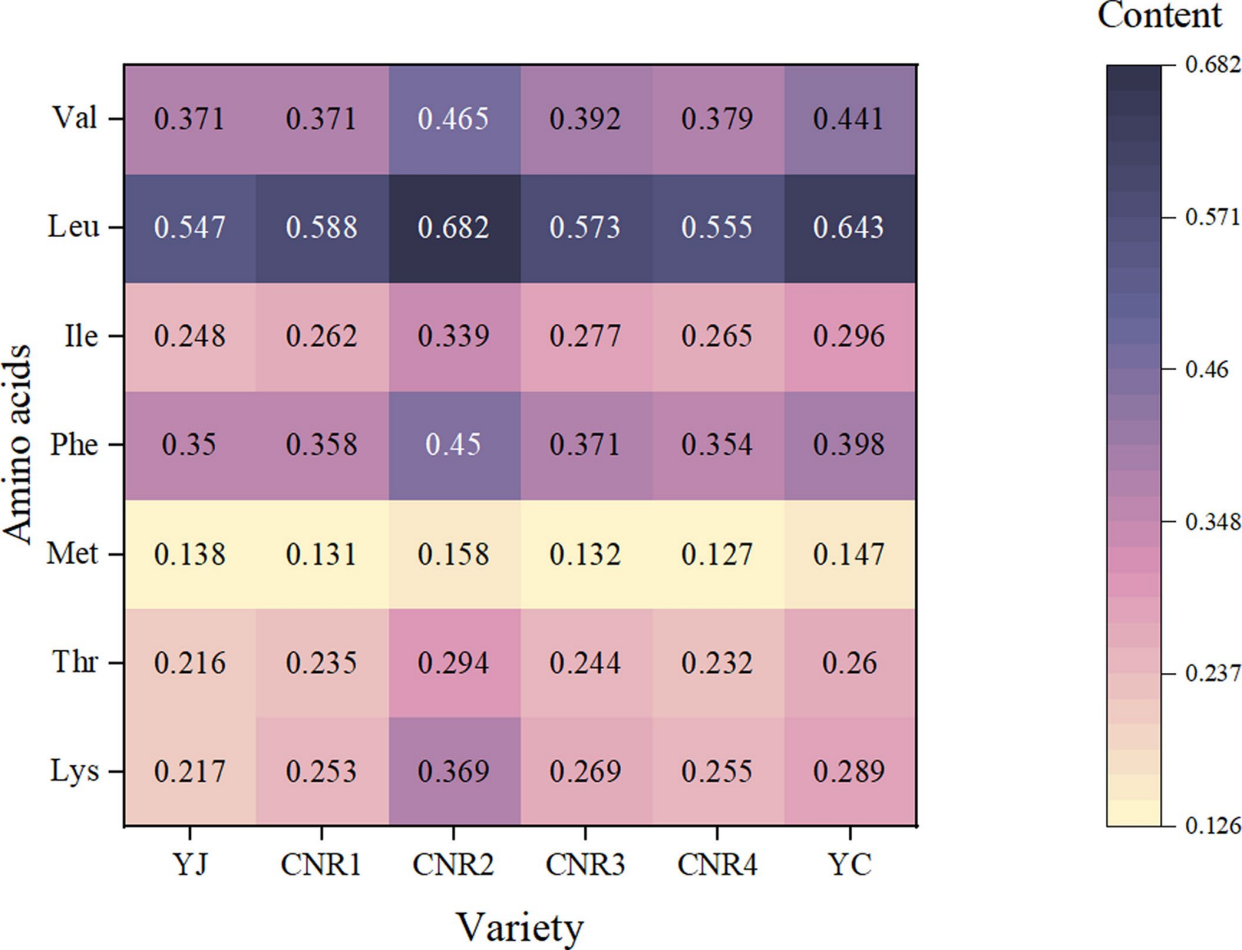
Essential amino acid contents of the six rice samples (g/100 g).

In rice, there are 20 major amino acids, including 8 essential amino acids that cannot be synthesized by the human body: lysine (Lys), threonine (Thr), methionine (Met), tryptophan (Trp), phenylalanine (Phe), isoleucine (Ile), leucine (Leu), and valine (Val) (23). According to a previous study (24), lysine, threonine, and methionine are regarded as the first, second, and third limiting essential amino acids in rice. Lysine is a major indicator of rice quality (25), as lysine can limit the absorption and utilization of other amino acids and proteins, resulting in nutritional deficiencies in humans. Therefore, the overall nutritional value of rice can be increased if the lysine content in rice is increased (26). In this study, all seven essential amino acids were detected by the amino acid analyzer, except for tryptophan (see Figure 1). YC, as brown rice, had a much higher content of all seven essential amino acids than YJ, indicating that most of the nutrients in rice were retained in the skin and germ parts, and that the excessive polishing of refined rice led to a serious loss of nutrients. In particular, the content of lysine—one of the main indicators of rice quality—was much higher in CNR2 than in the other samples. Therefore, through the above data, we believe that CNR2 has high nutritional value.

### 3.2 Starch content

As shown in Table 2, the values of apparent amylose content (AAC) of all samples are low (<20%). It was generally believed that the apparent amylose content is related to the taste quality, and the higher the AAC, the worse the taste (15). In this study, CNR2 had a significantly higher AAC than the other five samples, while YC had the lowest apparent amylose content. And it was found that the AAC values of compound nutritional rice were all higher than those of common rice. The higher AAC might be due to the degradation of most of the amylopectin into amylose or dextrin after the starch had been treated at high temperature and pressure which, in turn, led to higher final apparent amylose content (27).

**Table 2:** Apparent amylose content and resistant starch content of the six rice samples.

Samples	Apparent amylose content (%) AAC (%)	Resistant starch content (%) RS (%)
YJ	16.02 ± 0.29d	0.39 ± 0.06a
CNR1	18.08 ± 0.06b	0.17 ± 0.01b
CNR2	19.45 ± 0.33a	0.17 ± 0.01b
CNR3	16.42 ± 0.03d	0.17 ± 0.01b
CNR4	17.47 ± 0.41c	0.17 ± 0.02b
YC	11.83 ± 0.37e	0.39 ± 0.02a

Data are shown as the mean ± standard error of triplicate measurements. Different letters are followed after standard deviation to express significantly different (*p* < 0.05).

Based on digestive properties, starch can be classified as rapidly digestible starch (RDS), slowly digestible starch (SDS), and resistant starch (RS) (28). Among them, RS is considered a starch fraction that cannot be directly digested and absorbed in the small intestine, which means that the body cannot directly convert RS into soluble sugars for absorption in the body to achieve glycemic control (29). The RS content of common rice was low, about 1% or less; and currently cultivated highly resistant rice can have an RS content of up to about 10%. It was generally believed that staple foods with high RS content were more suitable for diabetic patients.

In this study, the RS content in the four experimental samples was significantly lower than that of YJ and YC. After analysis, we believe that there may be two reasons for this: first, the RS content in the four types of compound nutritional rice raw materials was too low, resulting in the overall RS content not significantly increasing after being extruded into rice; or, during the extrusion and the temperature was too high, causing excessive starch pasting and resulting in low RS content. Generally speaking, the higher the degree of starch pasting, the easier it is to be digested and absorbed, which means that the RS content would decrease instead of increase.

Based on the data we obtained, we believed that in order to obtain compound nutritional rice with high RS content, firstly, we should choose rice flour with high RS as the raw material, and secondly, we should appropriately lower the extruding temperature, which could reduce the possibility of over-pasteurization of starch as a means of increasing the RS content of the final product.

### 3.3 Starch crystal structure

**Figure 2:**
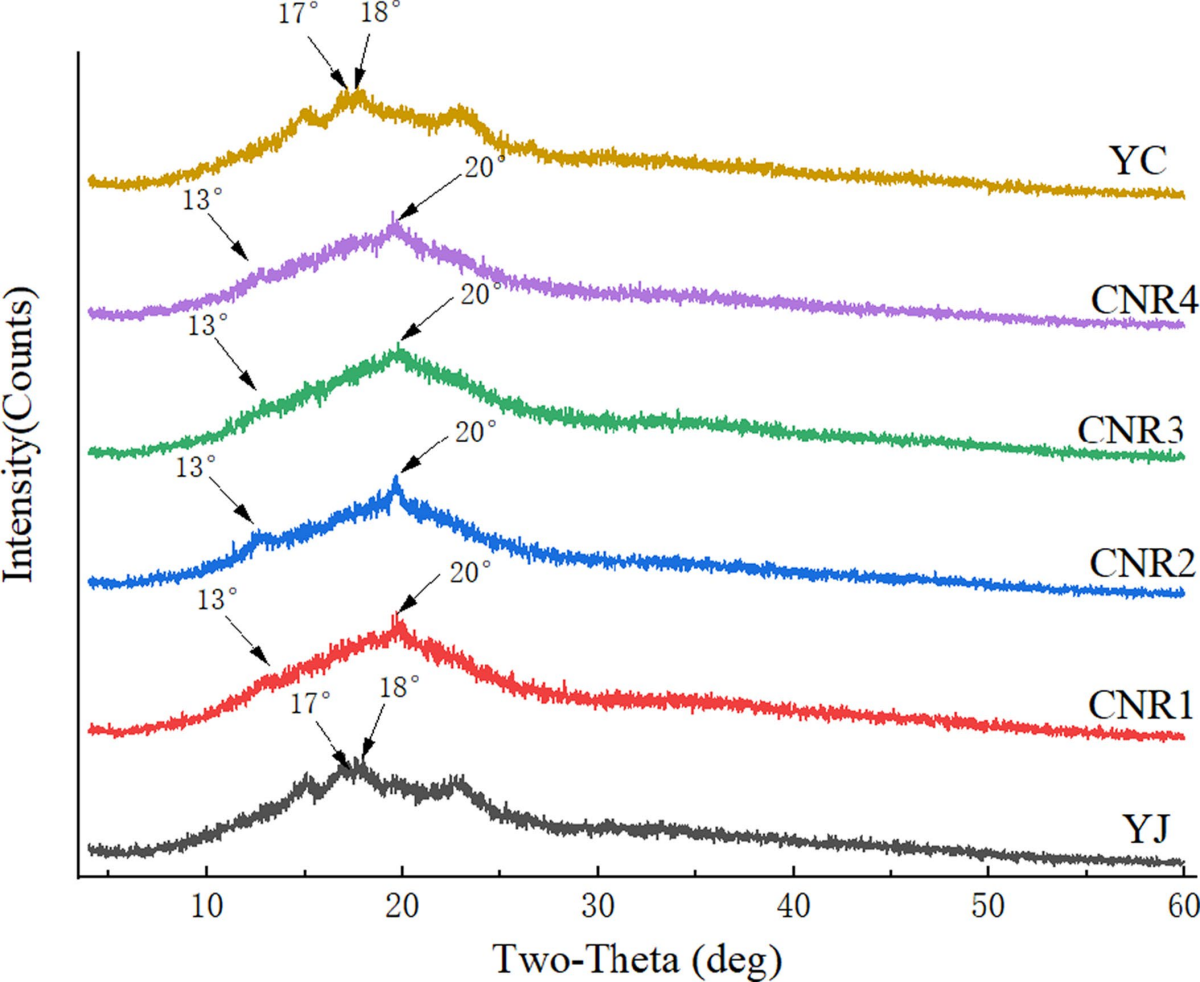
X-ray diffraction patterns of rice starch of the six rice samples.

In natural starch, the common crystalline structures are mainly type A and type B, both of which are double helix structures (30). The starch in common rice showed a typical A-type structure, having strong diffraction peaks near 15°, 17°, 18°, and 23°, with the diffraction peaks near 17° and 18° being connected double peaks. When the starch was treated by high-temperature extrusion, it was pasteurized, its crystalline structure was destroyed, and its crystallinity was reduced (31). In the diffraction pattern, the strong characteristic diffraction peaks disappeared and new strong diffraction peaks appeared around 13° and 20° (Figure 2). The reason for this was that, under high temperature and pressure, the original double helix structure of starch disintegrated into a single helix and recombined with lipids and alcohols to form a new starch-lipid complex, which is known as V-shaped crystallization (32).

In the present study, YJ and YC were common rice, while the remaining four samples were compound nutritional rice that underwent high-temperature extrusion operations. As shown in Figure 2, the diffraction patterns of YJ and YC exhibited a typical A-type structure, while the diffraction patterns of the four others models represented a change in starch structure to V-type. This conclusion is consistent with the previous literature and confirms that the structural function of starch changes under high temperature and pressure (33). However, there was a contradiction between the change in structure here and the difference in RS content. It is generally believed that the creation of the V-shaped structure of starch leads to an increase in RS content (34), which was significantly lower in the compound nutritional rice than in the control group in this paper. In this regard, we determined that this was because the RS content of the raw material of the experimental groups was too low and, even if the starch structure was changed, it could not lead to a significant increase in the overall RS content of the compound nutritional rice. Therefore, it would be a good choice to choose rice with high RS content as the raw material to be improved.

### 3.4 FT-IR spectra analysis

**Figure 3:**
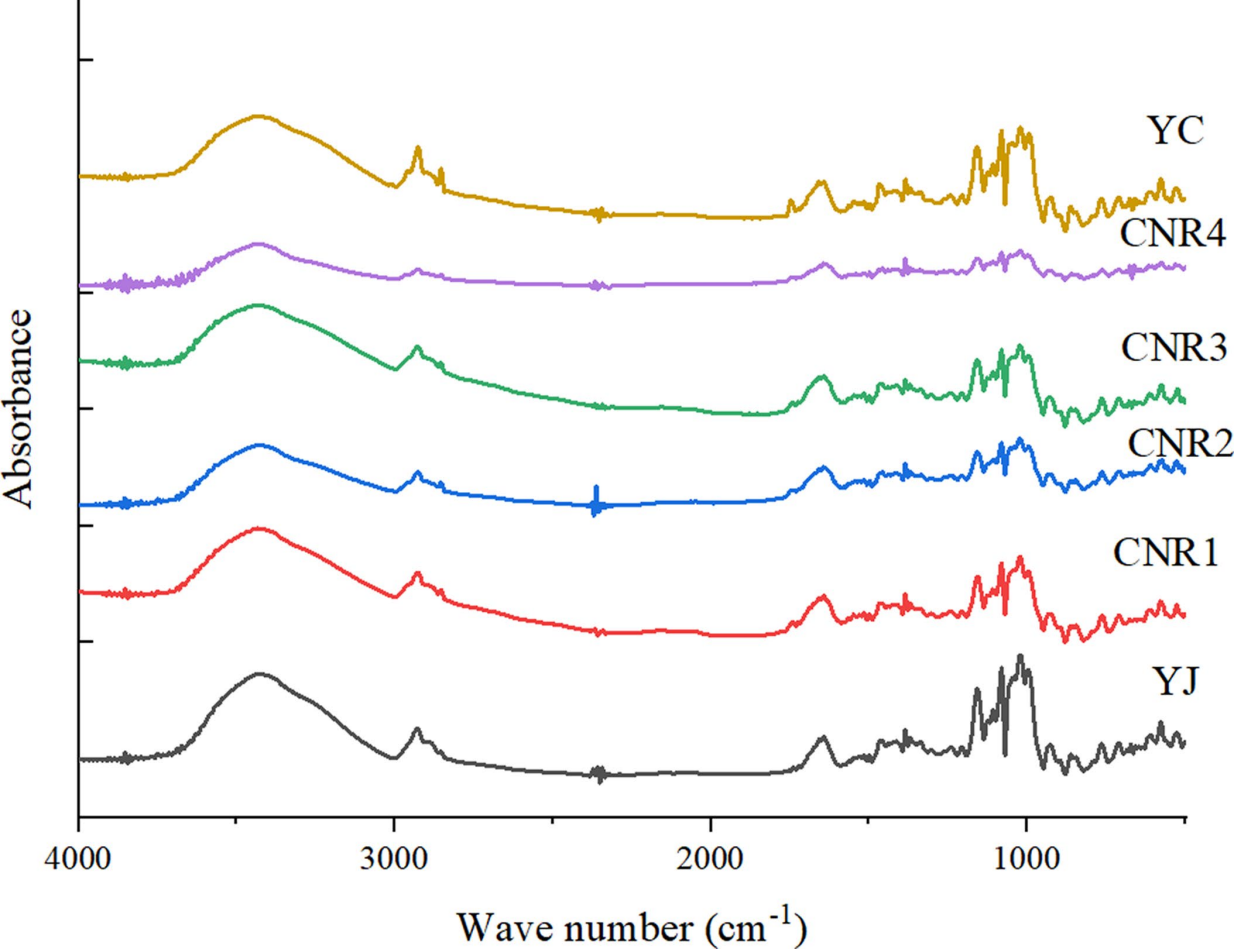
FT-IR spectra of the six rice samples.

According to a previous study (35), the absorption peaks in FTIR spectra mainly reflect the vibration of functional groups between starch molecules, and can determine the type of hydrogen bonding between starch molecules. The absorbance values in the 3,000–3,600 cm^−1^ band generally represented the vibration between starch hydrogen bonds. As shown in Figure 3, the comparison between the compound nutritional rice and the control group indicated a difference in the amplitude of changes in the 3,000–3,600 cm^−1^ band, and the amplitude of changes in the compound nutritional rice was significantly smaller than that for common rice, which might be due to the high temperature of the extrusion process of the compound nutritional rice causing hydrogen bonding interactions in the starch molecules (36).

According to previous studies (37), the absorption peaks at 1,022 cm^−1^ and 1,047 cm^−1^ represent the disordered and ordered structure of starch, respectively. Therefore, the absorbance ratio of 1,047 cm^−1^/1,022 cm^−1^ represented the ratio of ordered and disordered structures in starch (37), and it was generally believed that the greater the ratio, the higher the degree of order of starch. However, in this study, the ratio of ordered structure to disordered structure in multitrophic rice was not uniform. As shown in Supplementary Figure S1, the ratio of CNR1 and CNR3 was lower than that of the control group, while the ratio of CNR2 and CNR4 was higher. We believed that the difference in results was most likely due to the randomness of the selection of raw ingredients in the four types of CNR1 to CNR4. This also confirmed the necessity of standardization for the development of compound nutritional rice raw materials in the future.

### 3.5 Water absorption index and water solubility index

When rice was treated at a high temperature, its starch structure was disrupted, making it more capable of binding to water (35). From the data in Table 3, it can be seen that the WAI of the four compound nutritional rice samples was significantly higher than that of the common rice, this result also verified the above conclusion. From Table 2, we can see that the WSI of the four experimental samples was significantly higher than that of the control group YJ. The reason for this was that high temperatures degrade part of the large starch granules into small molecule products, which were more soluble in water, thus increasing the WSI value (14, 35). It was also possible that the elevated WSI and WAI might be responsible for changes in the protein network. However, due to the differences in raw materials between the compound nutritional rice, it was necessary to subsequently verify this conclusion by controlling the variables.

**Table 3:** Water solubility index and water absorption index of the six rice samples.

Samples	Water absorption index (WAI)	Water solubility index (WSI)
YJ	2.4018 ± 0.056d	0.0106 ± 0.009c
CNR1	5.5026 ± 0.255c	0.1108 ± 0.034a
CNR2	6.0242 ± 0.086ab	0.0602 ± 0.0004b
CNR3	5.7638 ± 0.165bc	0.0677 ± 0.020b
CNR4	6.1509 ± 0.176a	0.1249 ± 0.011a
YC	2.2701 ± 0.006d	0.0430 ± 0.001b

Data are shown as the mean ± standard error of triplicate measurements. Different letters are followed after standard deviation to express significantly different (*p* < 0.05).

### 3.6 Starch pasting properties as determined by RVA analysis

As can be seen from Table 4, the RVA values of the four types of compound nutritional rice were significantly lower than those of the control group, except for peak time (PT). The PT value of CNR2 was significantly higher than that of the control group YJ, but had no significant difference with the control group YC; while CNR1, CNR3 and CNR4 had significantly lower values than YC. It can be seen from the data in Table 3 that the change trends of peak viscosity (PV), trough viscosity (TV) and the cool-paste viscosity (CPV) between six samples were consistent, and the results of the four types of compound nutritional rice are significantly lower than those of common rice. Among them, PV, to a certain extent, expresses the water absorption and swelling capacity of starch. The PV of the four types of experimental group was significantly lower than the control group, mainly because the high-temperature extrusion process greatly damaged the starch granule structure and caused starch pasting, which, in turn, significantly lowered the PV of the compound nutritional rice. Generally speaking, rice with good steamed taste quality exhibits high breakdown (BD) and low setback (SB). When comparing the four experimental samples, CNR1 had relatively good taste quality, while that of CNR2 was relatively poor. However, compared with the control group, the taste quality of the four compound nutritional rice was much lower than that of common rice.

**Table 4:** Starch pasting properties of the six rice samples.

Samples	PV	TV	BD	CPV	SB	PT
YJ	3,295 ± 8.3a	1868 ± 21.4a	1,427 ± 29.1a	3,331 ± 35.1a	1,462 ± 14.0b	5.73 ± 0bc
CNR1	389 ± 2.1d	217 ± 2.5de	173 ± 3.1c	408 ± 2.1c	191.7 ± 0.6 cd	4.89 ± 0.03d
CNR2	428 ± 4.0c	375 ± 4.5c	53 ± 1e	533 ± 8.0b	158 ± 3.6d	6.04 ± 0.1a
CNR3	390 ± 22.5d	255 ± 24.8d	135 ± 5.3d	477 ± 49.7bc	222 ± 26.6c	5.95 ± 0.04ab
CNR4	367 ± 29.8d	209 ± 31.9e	159 ± 2.5 cd	432 ± 72.1c	223 ± 40.2c	5.67 ± 0.29c
YC	2,188 ± 14.1b	1,380 ± 27.1b	808 ± 22.3b	3,263 ± 42.3a	1883 ± 17.3a	6.07 ± 0.07a

Data are shown as the mean ± standard error of triplicate measurements. Different letters are followed after standard deviation to express significantly different (*p* < 0.05). The full names of the abbreviations are, respectively, peak viscosity (PV), trough viscosity (TV), cool-paste viscosity (CPV), breakdown (BD), low setback (SB), peak time (PT).

### 3.7 Steamed food quality analysis

It can be seen from the data in the Table 5 that there was a great difference between the taste quality of compound nutritional rice and common rice. In this experiment, the hardness, viscosity and elasticity of six samples had different changing trends. The hardness of CNR2 modified by the formula was significantly higher than that of the other 5 samples, but the difference between the other 5 samples was not significant. The viscosity values of the four compound nutritional rice varieties were significantly lower than YJ, the viscosity values of CNR2 and CNR4 were significantly lower than that of YC, and the viscosity values of CNR1 and CNR3 were not significantly different from that of YC. In terms of taste value, the score of YJ was the highest, the score of compound nutritional rice was significantly lower than that of YJ, while the score of CNR2 was the lowest and the taste value was the worst. The taste value was related to hardness, viscosity and elasticity. In general, the lower the hardness, the higher the elasticity, the higher the viscosity, and the higher the taste worth scoring. Although the elasticity of compound nutritional rice was better than common rice, the hardness of them was high and the viscosity was poor, resulting in the overall taste quality of compound nutritional rice was not as good as common rice.

**Table 5:** Steamed food quality of the six rice samples.

Samples	Hardness	Viscosity	Taste score
YJ	1.2289 ± 0.09b	0.1344 ± 0.02a	78.6 ± 1.59a
CNR1	1.2778 ± 0.16b	0.0789 ± 0.01b	75.3 ± 0.61b
CNR2	1.7489 ± 0.09a	0.0133 ± 0c	64.2 ± 0.5d
CNR3	1.3411 ± 0.20b	0.0811 ± 0.10b	72.7 ± 0.81b
CNR4	1.5078 ± 0.16ab	0.0178 ± 0.002c	65 ± 0.38d
YC	1.5767 ± 0.40ab	0.0933 ± 0.03b	67.7 ± 3.23c

Data are shown as the mean ± standard error of triplicate measurements. Different letters are followed after standard deviation to express significantly different (*p* < 0.05).

### 3.8 Artificial tasting analysis

**Figure 4:**
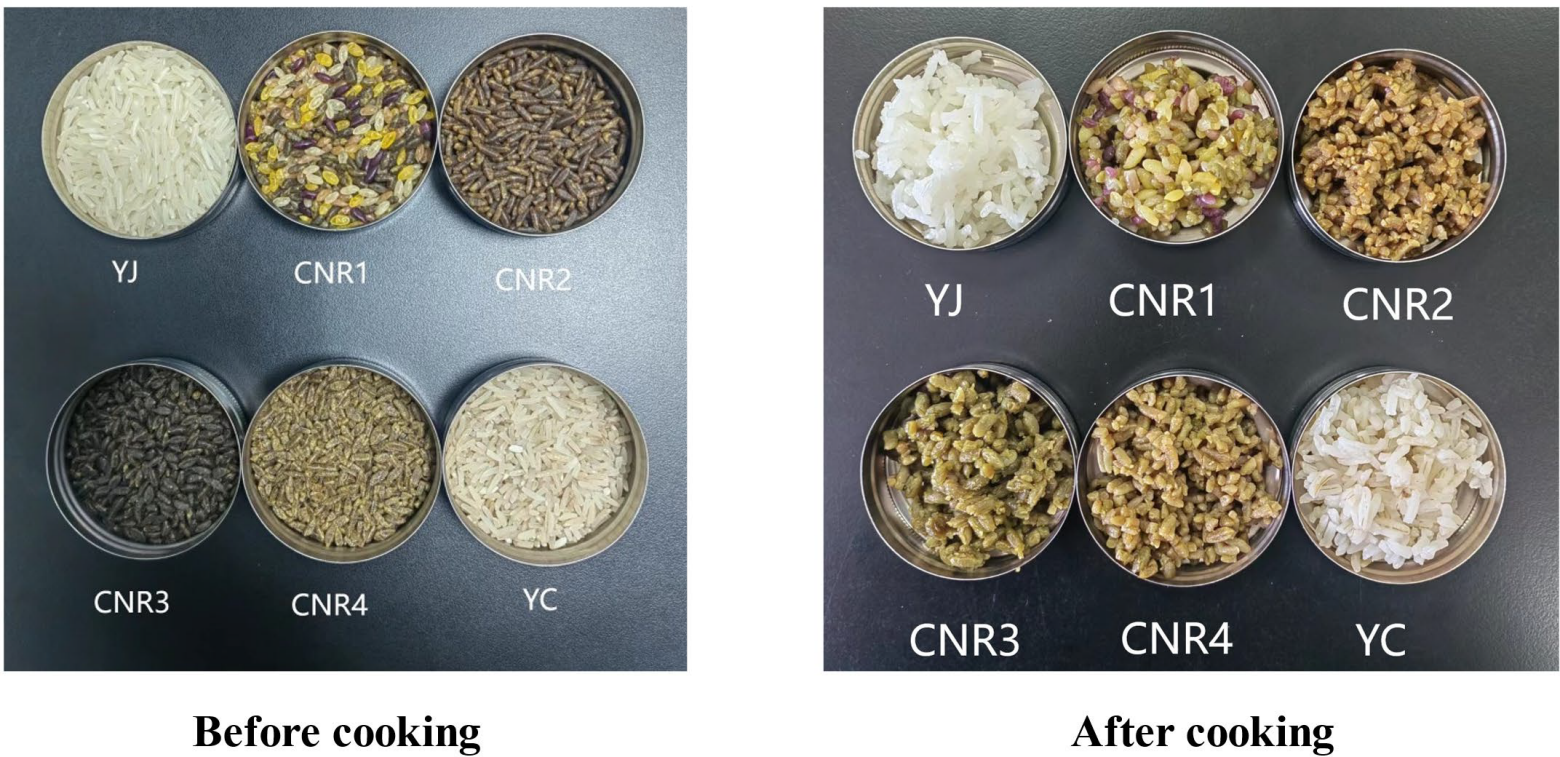
Comparative photos of six samples before and after cooking.

The specific evaluation of manual tasting is shown in Table 6. The four kinds of compound nutritional rice were all added with bitter melon and other nutritive powders, which made the compound nutritional rice not have the fragrance of ordinary rice after cooking, and the odor fraction was much lower than YJ and YC. As shown in Figure 4, the color of compound nutritional rice was varied, but its color would be deepened after cooking, and most of the tasters believed that the change of color will affect the appetite and thus reduce the evaluation of it. In addition to CNR4, the luster of the other 3 kinds of compound nutrition rice was significantly better than that of YC; After cooking, except for CNR1, the other 3 kinds of compound nutrition rice showed different degrees of rice explosion, and the integrity of CNR2 was the worst. The stickiness, softness and elasticity of compound nutritional rice were significantly lower than that of YJ, and the stickiness was significantly lower than that of YJ and YC. Moreover, the taste scores of the four kinds of compound nutritional rice were all lower than that of YJ and YC, and most of the tasters believed that the compound nutrition rice did not have the sweet taste of common rice when chewed, and some even had bitter taste. After the rice became cold, the compound nutritional rice appeared to be clagged and hardened, and the texture of the cold rice was significantly worse than that of common rice.

**Table 6:** Artificial tasting results of the six rice samples.

Samples	Smell	Color	Glossiness	Integraty	Stickiness	Elasticity	Hardness	Taste	Texture of cold rice	Total score
YJ	19.00 ± 0.82a	6.25 ± 0.50a	7.50 ± 0.58a	4.50 ± 0.57a	8.50 ± 0.58a	8.75 ± 0.50a	8.75 ± 0.48	22.00 ± 1.41a	4.00 ± 0.00a	89.25 ± 1.89a
CNR1	13.50 ± 0.58c	4.00 ± 0.00bc	7.75 ± 0.50a	4.25 ± 0.50ab	5.00 ± 0.82c	7.75 ± 0.40a	6.5 ± 0.58	16.00 ± 0.82b	3.25 ± 0.50b	68.00 ± 2.94c
CNR2	12.00 ± 0.00d	3.25 ± 0.50 cd	5.00 ± 1.15bc	2.25 ± 0.50c	3.50 ± 0.58d	4.75 ± 0.96bc	5 ± 0.82	14.50 ± 0.58c	1.75 ± 0.50d	52.00 ± 2.45e
CNR3	13.25 ± 0.50c	3.50 ± 0.58 cd	6.00 ± 0.82b	3.50 ± 0.58b	3.75 ± 0.50d	5.75 ± 0.96b	6 ± 0.82	15.00 ± 0.75bc	2.50 ± 0.58c	59.25 ± 2.36d
CNR4	12.75 ± 0.50 cd	3.00 ± 0.82d	4.25 ± 0.50c	2.50 ± 0.48c	4.00 ± 0.82d	3.75 ± 0.50c	5.75 ± 0.50	15.00 ± 0.82bc	1.75 ± 0.50d	52.75 ± 2.63e
YC	17.25 ± 0.96b	4.50 ± 0.58b	4.25 ± 0.50c	4.75 ± 0.50a	6.5 ± 0.58b	5.75 ± 0.50b	6.5 ± 0.58	18.75 ± 0.96b	3.25 ± 0.50b	71.50 ± 0.58b

Data are shown as the mean ± standard error of triplicate measurements. Different letters are followed after standard deviation to express significantly different (*p* < 0.05).

Based on the above indicators, YJ had the best sensory evaluation with a total score of 89.25, followed by YC with a total score of 71.50. The total scores of the four kinds of compound nutritional rice were all lower than 70 points, among which CNR1 was the highest with 68 points, and CNR2 was the lowest with 52 points. According to the results of artificial tasting, the taste of compound nutritional rice was much lower than that of common rice, and even some indexes are lower than YC. Most of the appraisers of the compound nutritional rice were that the smell was not as fragrant as that of common rice, the stickiness was not enough after the entrance, and there was a certain elasticity, but the taste was more slimy. It had a bitter taste after chewing, and the texture of the rice hardens as it cools. Therefore, the taster believes that the taste of compound nutritional rice needed to be further improved, and there was a large gap between it and common rice.

## 4 Discussion

Through our research results, we found that if the compound nutritional rice wants to develop for a long time, the company should vigorously improve its nutrition imbalance and poor taste quality in the future. Just like Nengfeng Biotechnology Co., Ltd., has focused on adjusting the selection and proportion of raw materials in the production of compound nutritional rice. The raw materials of rice flour with higher nutrition have been replaced and the amount of raw materials affecting the structure and taste of the finished product, such as bitter gourd and soybean, have been reduced. This is coupled with an improved extrusion process, which has enhanced the nutritional balance of the compound nutritional rice and its palatability (unpublished date).

## 5 Conclusion

In this study, we chose high-quality rice as the control group, hoping to understand the gap between high-quality and compound nutritional rice, hoping that compound nutritional rice will gradually become better tasting and nutritionally balanced in the future. The contents of protein, lysine and apparent amylose in compound nutritional rice were significantly higher than those in common rice. There was no significant difference in dietary fiber content, but GABA content was significantly lower than that of ordinary rice. After high temperature extrusion, the crystal structure of starch changed from A-type to V-type, and the proportion of ordered and disordered structure of starch also changed. The water absorption index and water solubility index of starch increased, and the cooking quality and taste quality of compound nutritional rice were obviously inferior to that of ordinary rice through machine scoring and manual tasting. In the RVA spectrum (except PT), other variables (such as BD and SB) were significantly lower than that of common rice.

To sum up, it was found that the compound nutritional rice had the characteristics of unreasonable nutrition, unbalanced nutrition and poor palatability. Due to the random selection of raw material varieties, it is impossible to guarantee the high nutritional value of the selected raw materials, resulting in nutritional imbalance. In addition, the poor palatability of some raw materials and the operation of high temperature extrusion will also lead to poor cooking taste quality. In the follow-up study, we suggest that the formulation of raw material selection criteria to improve nutrition from the source, so that the compound nutritional rice to achieve a high nutritional value and nutritional balance. Reducing the temperature of extrusion process as much as possible is also one of the ways to improve the taste value of compound nutritional rice. In the future, the developers of compound nutritional rice should pay more attention to the common progress of nutrition and taste, and establish a unified standard to judge whether the compound nutritional rice meets the requirements of high nutrition.

## Data availability statement

The original contributions presented in the study are included in the article/Supplementary material, further inquiries can be directed to the corresponding authors.

## Author contributions

DZ: Writing – original draft, Writing – review & editing. JL: Conceptualization, Resources, Writing – review & editing. DY: Methodology, Writing – original draft. JW: Project administration, Supervision, Writing – review & editing. QL: Writing – original draft. HS: Methodology, Writing – original draft. MH: Data curation, Methodology, Writing – original draft. FM: Data curation, Investigation, Writing – original draft. YZ: Formal analysis, Investigation, Writing – original draft. XL: Supervision, Writing – review & editing. YS: Resources, Supervision, Writing – review & editing. DL: Resources, Supervision, Writing – review & editing. BB: Resources, Supervision, Writing – review & editing.
