# Supplementary material for: Differences in cooking taste and physicochemical properties between compound nutritional rice and common rice
Source: Front Nutr. 2024 Jul 31;11:1435977. doi: 10.3389/fnut.2024.1435977 (PMC11322078; doi:10.3389/fnut.2024.1435977)
Supplement: Supplementary file 1 [file Data_Sheet_1.docx]

**Supplementary Table 1.** Compound Nutritional Rice Specific Ingredients Table

| NO. | raw material component | | | | | | | |
| --- | --- | --- | --- | --- | --- | --- | --- | --- |
|  | basic raw material（~70%） | | Cereal crop flour  （~10%） | | potato crop flour（~10%） | vegetable flour（~5%） | legume flour and herbal powder (~5%) | other nutritional powders |
| CNR1 | | rice flour | millet flour, corn flour, buckwheat flour,  oat flour, quinoa flour | purple yam flour, sweet potato flour, potato flour | | Bitter melon powder, pumpkin powder, yam powder, kudzu powder, konjac powder | Mung bean powder, Cordyceps pupa powder, Wolfberry powder, soybean protein powder, Poria powder | mulberry leaf extract, edible refined salt |
| CNR2 | | rice flour | millet flour, corn flour, buckwheat flour, quinoa flour, oat flour，wheat flour | purple yam flour, sweet potato flour, potato flour | | Spinach Powder, Cucumber Powder, Shiitake Mushroom Powder, Bitter Melon Powder, Pumpkin Powder, Yam Powder, Pueraria Mirifica Powder, Konjac Powder | Mung Bean Powder, Soybean Powder, Chrysanthemum Cordyceps Powder, Wolfberry Powder, Poria Powder, | Inulin, Luo Han Guo powder, Mulberry leaf extract, edible refined salt |
| CNR3 | | rice flour | millet flour, corn flour, buckwheat flour, quinoa flour, oat flour，wheat flour | purple yam flour, sweet potato flour, potato flour | | Spinach Powder, Lily Powder, Cucumber Powder, Shiitake Mushroom Powder, Bitter Melon Powder, Pumpkin Powder, Yam Powder, Pueraria Mirifica Powder, Konjac Powder | Mung Bean Powder, Soybean Powder, Chrysanthemum Cordyceps Powder, Wolfberry Powder, Poria Powder | Inulin, Luo Han Guo powder, Mulberry leaf extract, edible refined salt |
| CNR4 | | rice flour | Millet flour, corn flour, buckwheat flour, Highland barley flour, oat flour, quinoa flour | purple yam flour, sweet potato flour, potato flour | | Bitter melon powder, pumpkin powder, yam powder, kudzu powder, Konjac powder | Mung Bean Powder, Chrysanthemum Cordyceps Powder, Wolfberry Powder, Poria Powder | mulberry leaf extract, edible refined salt |

**Supplementary Table 2** **Specific Scoring Rules for Taste Tasting**

| Level 1 indicator score | Level 2 indicator score | Specific Characterization Score |
| --- | --- | --- |
| Smell  20 points | Purity, intensity  20 points | With the unique aroma of rice, fragrant; 18 ~ 20 points |
|  |  | With the unique aroma of rice, rice fragrance; 15 ~ 17 points |
|  |  | With the unique aroma of rice, not obvious; 12 ~ 14 points |
|  |  | No smell, but no odor; 7 ~ 12 points |
|  |  | Rice smells bad; 0 ~ 6 points |
| Appearance  20 points | color  7 points | White in color; 6 ~ 7 points |
|  |  | Normal color; 4~5 points |
|  |  | Brown or grey rice; 0 ~ 3 points |
|  | glossiness  8 points | Clearly glossy; 7 ~ 8 points |
|  |  | Slightly glossy; 5 ~ 6 points |
|  |  | No gloss; 0~4 points |
|  | Rice integrity  5 points | Tightly structured rice with good grain integrity; 4 ~ 5 points |
|  |  | Mostly tightly structured and intact; 3 points |
|  |  | Broken rice grains; 0 ~2 points |
| Palatability  30 points | Stickiness  10 points | Smooth, tacky, not sticky: 8 ~ 10 points |
|  |  | Sticky, basically non-stick teeth: 6 ~ 7 points |
|  |  | Sticky, sticking to teeth; or non-sticky: 0 ~ 5 points |
|  | Elasticity  10 points | Chewy rice: 8 ~ 10 points |
|  |  | Slightly chewy rice: 6 ~ 7 points |
|  |  | Fluffy, hard rice, feels crumbly: 0 ~ 5 points |
|  | Hardness  10 points | Moderate softness: 8 ~ 10 points |
|  |  | Feels slightly hard or slightly soft: 6 ~ 7 points |
|  |  | Feels very hard or soft: 0 ~ 5 points |
| Taste  25 points | Purity, durability  25 points | Chewing with a fresher and sweeter flavor: 22 ~ 25 points |
|  |  | Chewing with light refresh and sweet flavor: 18 ~ 21 points |
|  |  | No light flavor or sweetness, but no off-flavor: 16 ~ 17 points |
|  |  | No light flavor or sweetness, but off-flavor：0～15 points |
| Cold rice texture  5 points | Agglomeration, viscoelasticity, hardness  5 points | Looser, better viscoelasticity, moderate hardness: 4 ~ 5 points |
|  |  | Clumping, slightly poor viscoelasticity, slightly hardened: 2 ~ 3 points |
|  |  | Hardening, poor viscoelasticity, hard: 0 ~ 1 points |


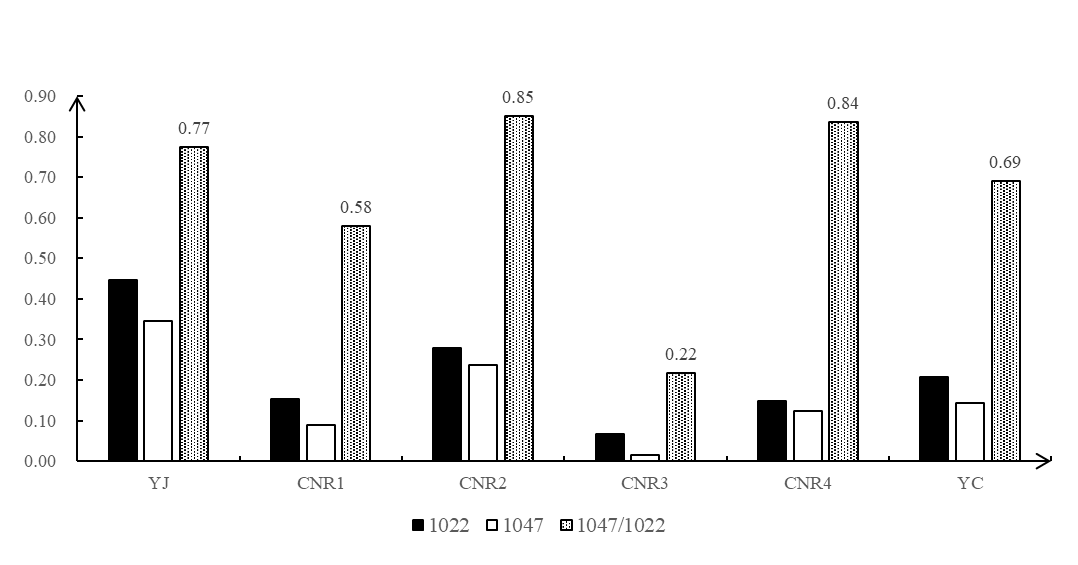


**Supplementary Figure 1.** Ratio of ordered to disordered structures in starch of the six samples
